# Embryonic miRNA Profiles of Normal and Ectopic Pregnancies

**DOI:** 10.1371/journal.pone.0102185

**Published:** 2014-07-11

**Authors:** Francisco Dominguez, Juan Manuel Moreno-Moya, Teresa Lozoya, Ainhoa Romero, Sebastian Martínez, Mercedes Monterde, Marta Gurrea, Blanca Ferri, Maria Jose Núñez, Carlos Simón, Antonio Pellicer

**Affiliations:** 1 Fundación IVI, Instituto Universitario IVI, INCLIVA, Valencia, Spain; 2 Hospital Universitario LaFe, Valencia, Spain; 3 INCLIVA Biomedical Research, Valencia, Spain; Ospedale Pediatrico Bambino Gesù, Italy

## Abstract

Our objective was to investigate the miRNA profile of embryonic tissues in ectopic pregnancies (EPs) and controlled abortions (voluntary termination of pregnancy; VTOP). Twenty-three patients suffering from tubal EP and twenty-nine patients with a normal ongoing pregnancy scheduled for a VTOP were recruited. Embryonic tissue samples were analyzed by miRNA microarray and further validated by real time PCR. Microarray studies showed that four miRNAs were differentially downregulated (hsa-mir-196b, hsa-mir-30a, hsa-mir-873, and hsa-mir-337-3p) and three upregulated (hsa-mir-1288, hsa-mir-451, and hsa-mir-223) in EP compared to control tissue samples. Hsa-miR-196, hsa-miR-223, and hsa-miR-451 were further validated by real time PCR in a wider population of EP and control samples. We also performed a computational analysis to identify the gene targets and pathways which might be modulated by these three differentially expressed miRNAs. The most significant pathways found were the mucin type O-glycan biosynthesis and the ECM-receptor-interaction pathways. We also checked that the dysregulation of these three miRNAs was able to alter the expression of the gene targets in the embryonic tissues included in these pathways such as GALNT13 and ITGA2 genes. In conclusion, analysis of miRNAs in ectopic and eutopic embryonic tissues shows different expression patterns that could modify pathways which are critical for correct implantation, providing new insights into the understanding of ectopic implantation in humans.

## Introduction

Ectopic pregnancy (EP) is an early pregnancy complication in which a fertilized ovum implants outside the uterine cavity. Implantation may occur anywhere along the reproductive tract with the most common implantation site being the fallopian tube. The etiology of EP is not completely known, but it is probably due to retention of the embryo caused when the embryo-tubal environment is impaired, thus allowing tubal implantation to occur [1], [2]. EP affects approximately 1–2% of pregnant women, and may seriously compromise women's health and their future fertility as tubal rupture is a common complication [3]. In fact, hemorrhage from ectopic pregnancy is still the leading cause of pregnancy related maternal death in the first trimester and accounts for 4–10% of all pregnancy related deaths, despite the use of improved diagnostic methods leading to earlier detection and treatment [4], [5].

Recent evidence has conclusively demonstrated that the regulation of numerous key biological processes does not depend only on classical transcriptional mechanisms, and that other regulatory phenomena such as epigenetic mechanisms do have important roles. These epigenetic mechanisms include not only DNA methylation and the post-translational modification of histones, but also small non-coding RNAs, including microRNAs (miRNAs) [6], [7].

MiRNAs are expressed in a cell-specific manner [8] and function as negative gene expression regulators, controlling essential processes such as cellular differentiation, proliferation, and apoptosis [9]. They are processed by the ribonucleases Drosha and Dicer which gives rise to their mature form. Mature miRNAs are incorporated into the RISC (RNA-induced silencing complex) where they bind to the 3′UTRs of complementary mRNAs at the seed region; this induces target mRNA degradation or inhibits their translation, resulting in gene silencing [10]–[12]. Furthermore, these short and robust sequences can target hundreds of genes, altering gene expression within a particular tissue or even causing an altered condition such as in many types of cancer [13], [14]. MiRNAs have already been implicated in some regulatory processes such as the acquisition of endometrial receptivity [15]–[17] and decidualization [18]. Thus, our aim with this work was to discover the differential miRNA expression pattern of eutopic and ectopic embryonic tissues.

## Materials and Methods

### Ethical approval

This study was approved by the Institutional Review Board/Independent Ethics Committee of the *Hospital Universitario La Fe*, Valencia, Spain (2012/0340). Early embryonic tissue (mostly trophoblast) was collected after obtaining written informed consent from each patient for this research under the principles of the Declaration of Helsinki for medical research involving human subjects.

### Study design

Twenty-four patients suffering from EP and twenty-nine control patients were recruited for the study. The study design included a first series of eight patients suffering from tubal EP and eight patients scheduled for a voluntary termination of pregnancy (VTOP) for the microarray discovery study. To validate the differentially expressed miRNAs found using microarray technology we used real time PCR on samples from a second series of 15 EP and 21 VTOP patients. The characteristics of the patients included in series one and two are described in Tables 1 and 2 respectively.

**Table 1:** 

	Age	Gestational week	Gravidity	Number of births	History of previous spontaneous or induced abortions	Number of abortions	History of previous ectopic pregnancy	History of abdominal or pelvic surgery	History of infertility	IUD user or progestogen contraception
EP	30.75±1.78	7+1	3.37±0.99	0.5	4 (50%)	1.75	1 (12.5%)	3 (37.5%)	0	0
VTOP	26±3.17	7+6	2.12±0.29	0.5	3 (37.5%)	0	0	1 (12.5%)	1 (12.5%)	2 (25%)
P-VALUE	ns	0,027	ns	-	-	-	-	-	-	-

Patient characteristics for the samples used for the array studies. Data is presented as the mean plus or minus (±) standard error mean (SEM), or as the number and its corresponding percentage; ns  =  not significant. Gestational age (in days), gravidity, the number of births, history of previous spontaneous or induced abortions, and the number of induced abortions were evaluated with the Mann–Whitney U test, while the history of previous ectopic pregnancies, abdominal or pelvic surgeries, infertility, or use of an IUD or progestogen contraception was evaluated using Fisher's exact test.

**Table 2:** 

	Age	Gestational week	Gravidity	Number of births	History of previous spontaneous or induced abortions	Number of abortions	History of previous ectopic pregnancy	History of abdominal or pelvic surgery	History of infertility	IUD user or progestogen contraception
EP	30.81±1.55	6+6	2.19±0.31	0.31	7 (43,7%)	0.81	1 (6.25%)	4 (25%)	3 (18%)	0
VTOP	24.62±1.86	7+4	2.09±0.32	0.42	5 (23,8%)	0.23	1 (4%)	4 (19%)	0	15 (75%)
P-VALUE	0.002	ns	ns	-	-	-	-	-	-	-

The patient characteristics for the samples used for the real time PCR analysis. Data is presented as the mean plus or minus (±) the standard error mean (SEM) or as the number and its corresponding percentage; ns  =  not significant. Gestational age (in days), gravidity, the number of births, history of previous spontaneous or induced abortions, and the number of induced abortions were evaluated with the Mann–Whitney U test while any history of previous ectopic pregnancies, abdominal or pelvic surgeries, infertility, or use of IUD or progestogen contraception was evaluated using Fisher's exact test.

### Human samples

All the samples obtained from patients included in this study after signed informed consent (EP and VTOP) were properly dated according to the date of the last menstrual period (week of pregnancy). The diagnosis of EP was based on clinical and physical examination, transvaginal ultrasound, and serial quantitative β-hCG levels, and confirmed by laparoscopy in which the tissue was removed. The EP patients did not receive methotrexate treatment, and laparoscopy was performed as follows: ectopic pregnancies selected for this study were unruptured gestations located in the isthmus or the proximal ampulla. The tube containing the ectopic pregnancy was grasped at both sides (approximately 1 cm away from the gestation) and bipolar coagulation applied. Similarly, the adjacent mesentery was also coagulated. Then, salpingectomy was performed employing scissors. Then, a longitudinal antimesenteric incision into the surface of the tube and applied mild pressure with two fingers to extract the gestational sac. Embryonic tissue was carefully separated from obvious blood clots or tubal tissue in the operating room under a stereomicroscope and were immediately placed in TRIzol reagent (*see below*), frozen, and stored at −80°C until use. A piece of each sample was sent to the Pathology Department (*Hospital Universitario La Fe*), who provided histological confirmation of ectopic pregnancy and the absence of tubal tissue. The fetal dilation and evacuation method or fetal aspiration technique were performed in VTOP women to obtain the embryonic tissue.

### RNA extraction

All tissue samples were processed for total RNA extraction using the TRIzol reagent following the manufacturer's protocol (Invitrogen, CA). The quantity (ng/ml) and purity (260/280 and 260/230 ratio) of the RNAs obtained was measured using a NanoDrop 2000 spectrophotometer (Thermo-Scientific) and the presence, proportion, and quality of miRNAs were assessed with Small RNA LabChips and a BioAnalyzer (Agilent).

### MiRNA microarrays, principal component analysis, and clustering

To compare the pregnancy tissue miRNA profiles between VTOP and EP samples, human miRNA v3.0 8x15K microarrays (Agilent, Technologies Inc., DE, USA) that evaluate the expression of 866 human miRNAs were used (See GEO data for a complete list of these miRNAs). Total RNA from each sample was amplified, labeled, and hybridized according to Agilent's instructions [19]. Microarrays were scanned using the GenePix Pro scanner and the data obtained for each probe was analyzed. Following this, the intensity values were logarithmically and quartile normalized using R software and the Bioconductor libraries ‘limma’ and ‘tkrplot’. Principal component analysis (PCA) was used to represent the variance between samples on a 3D plot, reducing all the values of the differentially expressed miRNAs to three principal components (PC1, PC2, and PC3). Mean values for each probe were merged and the data was reduced to a PCA representation using an R script and the ‘prcomp’ package [20]. MeV software [21] was used for statistical analysis. Microarray data is available for public access in the GEO database with accession number GSE44731.

### Real time PCR validation

The microarray results were validated by real time PCR using miScript reverse transcription and miScript SYBR Green PCR commercial kits (Qiagen, Valencia, CA, USA) according to the manufacturer's protocols. Specific primer assays for hsa-miR-196b, hsa-miR-223, hsa-mir-30a, and hsa-mir-451 were purchased from Qiagen and real time PCR quantification was carried out in a LightCycler 480 (Roche). To normalize the expression values, a small nuclear RNA with constitutive expression, SNORD96A, was used as a housekeeping control [22]. Expression levels were quantitatively analyzed using the 2ΔΔCt method and were normalized to VTOP controls.

### Bioinformatic analysis of the target genes and functions that may be regulated by selected miRNAs

We performed computational analysis to identify genes and pathways that might be modulated by the altered-expression miRNAs that we identified. For the *in silico* analysis we used three different software programs: the web-based tool ‘DIANA-miRPATH’ together with microRNA.org [23], [24] to predict the pathways affected by potentially targeted genes; and ‘IPA’ software (Ingenuity Systems) to identify the biological functions and/or diseases that were most relevant to the data set. The gene expression of several target genes (ITGA2, GALNT7, GALNT13, GALNT1, and COL1A2) was measured by RT-PCR to compare their expression with their previously predicted deregulation (see above).

### JEG3 miRNA transfection

In vitro cultured trophoblast cell line JEG3 at 50% of confluency was transiently transfected with 50 nM of either miR-196b and miR-223 mimic or scramble miRNA using HiPerfect, following the manufacturer's instructions (Qiagen, Valencia, CA, USA); after 24, 48 and 72 h RNA was extracted from the cells. To ensure that the miRNA fraction was recovered we performed RNA extraction using the miRNeasy Kit (Qiagen). The RNA extracted was quantified using a NanoDrop spectrophotometer (Thermo Fisher Scientific, Inc., MA, USA) and the quality of RNA samples was assessed using a Nano LabChip BioAnalyzer 2100 (Agilent Technologies, Inc., DE, USA). Gene expression of predicted targets was performed by Real Time PCR. The experiments were repeated three times.

### MiScript qPCR for transfection efficiency

To evaluate the transfection efficiency, levels of miR-196b and miR-223 in JEG3 (compared with the housekeeping gene RNAU6-2) were measured by quantitative PCR with the miScript reverse transcription kit, miScript SYBR Green PCR kit and miScript primer assays according to the manufacturer's recommendations (Qiagen). In both cases miRNAs trasfected were increased between 1000 and 10.000 fold change compared to controls. Furthermore, approximately 95% of the cells transfected with scramble showed up fluorescence for GFP, meaning that the percentage of transfection was close to 100%.

### Statistical analysis

For microarray statistical analysis, MeV software was used. Samples were grouped into cluster hierarchies using Pearson correlation, and then they were subjected to PCA. Following this, a group variable was assigned to each sample and significance analysis of microarrays (SAM) analysis was used to estimate the changes in the expression level (fold change) of the miRNAs between the different samples. A false discovery rate (FDR) correction of less than 5% was used. For qPCR statistical analysis the expression levels of samples were subjected to a Mann-Whitney U test using SPSS v17.0 software. For biological process predictions the right-tailed Fisher's exact test was used to calculate a *p*-value.

## Results

### MiRNA microarray comparison of pregnancy-derived tissue from ectopic pregnancies and voluntary termination of pregnancy controls

**Figure 1:**
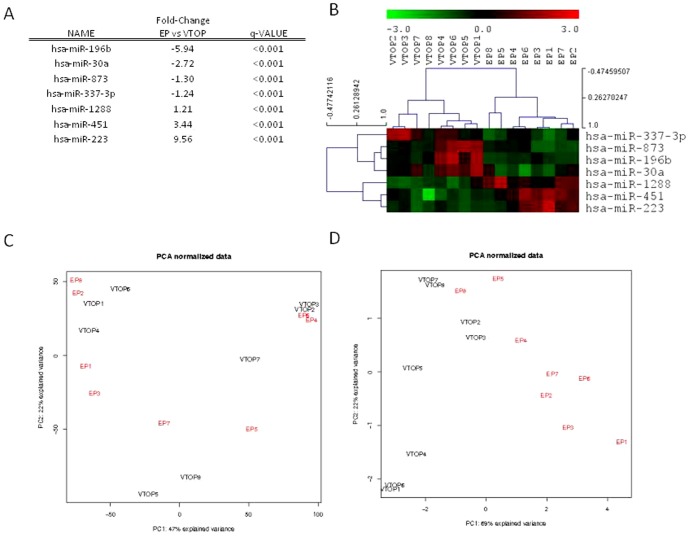
A list of differentially expressed miRNAs in embryonic tissues. Principal component analysis (PCA) and clustering analysis. (A) List of significantly altered miRNAs in pregnancy-derived tissues from ectopic pregnancies (EPs) compared to voluntary termination of pregnancy (VTOP) tissues using microarray analysis. Negative fold-change values represent downregulation and positive values represent upregulation. (B) Clustering and heat map analysis was performed using MeV software to cluster samples into hierarchies using Pearson correlation. All the samples classify well into two different populations (EP and VTOP) according to the expression of these seven miRNAs. The expression levels of these miRNAs were standardized and are represented as a heat map, showing variation ranging from high expression (red) to low expression (green) in each of the samples analyzed. (C) Three dimensional supervised PCA (which determines the key variables within the data set that explain the differences between the samples) for all the 16 tissue samples included in the microarray study, based on the expression profiles of the seven differentially expressed miRNAs. EP samples are clearly distinguished from VTOP sample sets at a separate position in the PCA analysis, and moreover, the EP samples and the VTOP samples clearly cluster together.

A total of 866 known miRNAs in first trimester embryonic tissue from eight EP patients were compared with the miRNAs from embryonic tissue derived from eight VTOP patients using miRNA microarrays. All 16 samples used in this microarray study passed extensive RNA and miRNA quality controls (see materials and methods). Among the 866 miRNAs screened in our microarray, the expression of seven of them were significantly different between pregnancy-derived tissue obtained from EPs and VTOPs after applying SAM with an FDR correction of less than 5% (Figure 1A). Four miRNAs (hsa-miR-196b, hsa-miR-30a, hsa-miR-873, and hsa-miR-337-3p) were found to be downregulated in EP versus healthy pregnancy tissues, and three miRNAs (hsa-miR-1288, hsa-miR-451, and hsa-miR-223) were upregulated in EP compared to control pregnancy tissue samples (Figure 1A). The miRNA downregulated by the highest amount was hsa-miR-196b, with a 5.94-fold change compared to VTOP tissue, and the miRNA with the most increased expression was hsa-miR-223 with a 9.56-fold change compared to VTOP controls.

To confirm that the results obtained from the array could separate EP and VTOP samples, we first applied a unsupervised PCA including all the miRNAs measured in the array experiment. (Fig1C). Then, a supervised PCA was applied to the seven differentially expressed miRNAs, showing a clear difference between them, indicating that embryonic tissue from EPs has a completely different and robust miRNA expression profile compared to those expressed in a normal ongoing pregnancy (Figure 1D). Moreover, both embryonic tissue sample types clustered into two separate populations (Figure 1B) according to the expression of these seven miRNAs.

### Validation of miRNAs in the embryonic tissue by real time PCR

**Figure 2:**
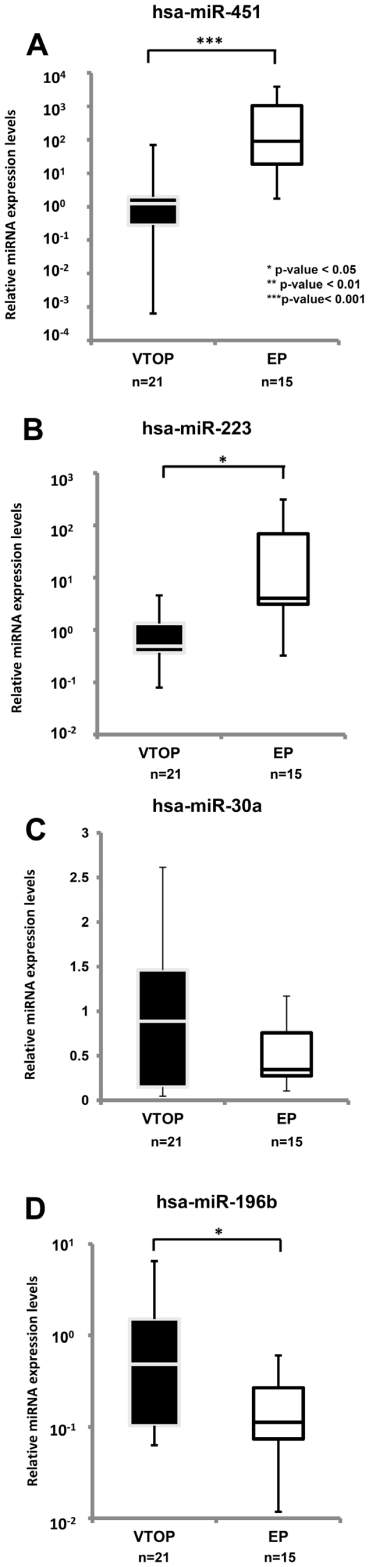
Validation of miRNAs in ectopic pregnancies and controls by real time PCR. 21 VTOP and 15 EP embryonic samples were used for the validation study. We observed a very significant increase (p<0.001) in the EP tissue samples compared to VTOP samples for hsa-miR-451(A) and hsa-miR-223 (B) expression, while hsa-miR-196b (D) showed a significant (p<0.05) downregulation compared to VTOP controls. No statistical differences in expression were found for hsa-miR-30a (C). All data are presented as relative miRNA expression levels. *p-value <0.05; **p-value <0.01; ***p-value <0.001. Box plots represent the first quartile, median and third quartile; error bars show maximum and minimum relative expression levels.

We first validated the miRNAs found in the microarray analysis by performing real time PCR on hsa-miR-196 and hsa-miR-223 in the same samples used for the microarray experiments (Figure S1). Then we validated the four miRNAs which had a more than two fold expression change in the microarray analysis by performing real time PCR in a larger cohort of samples We used 21 VTOP samples and 15 EP embryonic tissues for the validation studies. We confirmed a significant (*p*<0.001) increase in hsa-mir-451 (Figure 2A) and hsa-mir-223 (Figure 2B) miRNA expression (*p*<0.05) in EP samples compared to VTOP control samples, which was an even higher increase than in our microarray study. Significant (*p*<0.05) hsa-mir-196b downregulation was also detected in EP samples compared to controls in this set of samples (Figure 2D). However, hsa-mir-30a did not show any change in expression when we compared VTOP with EP samples (Figure 2C).

### Bioinformatic studies of miRNA targets and pathways

**Figure 3:**
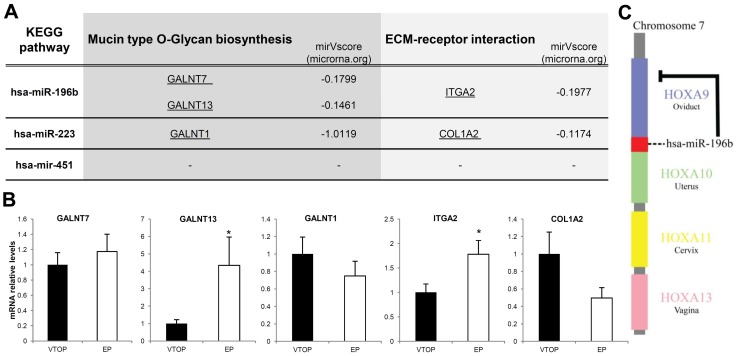
Predicted genes and gene validation. (A) The most significant KEGG pathways for mir-196b, 223, and 451 miRNAs, together with the targeted genes for each miRNA and their mirV score are shown. (B) Real time PCRs were performed on EP and VTOP embryonic tissues for each of the predicted target genes: GALNT7, GALNT13, GALNT1, ITGA2, and COL1A2. GALNT13 and ITGA2 were found to be significantly upregulated in EP samples compared to VTOP samples. (C) hsa-miR-196b is encoded in an intron of the HOXA9 gene and inhibits the expression of this gene.

As an initial approach to functionally studying the differential profile of the statistically different miRNAs identified in the EP and VTOP embryonic samples, we performed a computational analysis to identify the genes and pathways which might be modulated by the three differentially expressed miRNAs we had found: miR-196b, miR-223, and miR-451. For the *in silico* analysis, we used the DIANA-miRPATH software to analyze which gene targets and pathways the differentially expressed miRNAs found in EP samples might alter. We obtained various potentially affected pathways with different genes targeted by the miRNAs, suggesting that the altered expression profile of these miRNAs in EPs may affect *mucin biosynthesis*, *purine metabolism*, *ECM receptor interaction*, and *MAPK and ErbB signaling pathways*, among others (data not shown). The most significant pathways found were *the mucin type O-Glycan biosynthesis* and the *ECM-receptor-interaction* pathways. We found three different predicted genes which were altered in the mucin pathway: GALNT7 and GALNT13 (repressed by 196b), and GALNT1 (repressed by 223; Figure 3A). Another two genes, integrin A2 (ITGA2) and collagen alpha-2(I) chain (COL1A2), were also targeted by these two miRNAs. To check the ability of these miRNAs to alter the expression of predicted target genes, we performed real time PCR on all these target genes in our embryonic tissue samples. Significant upregulation was confirmed for GALNT13 and ITGA2 when we compared EP samples to controls (Figure 3B). However, we were unable to find any significant difference in the expression of GALNT7, GALNT1, or COL1A2, although the expected tendency towards upregulation was found in these three cases (Figure 3B).

### In vitro transfection of miR-196b and miR-223 in trophoblastic JEG3 cell line

**Figure 4:**
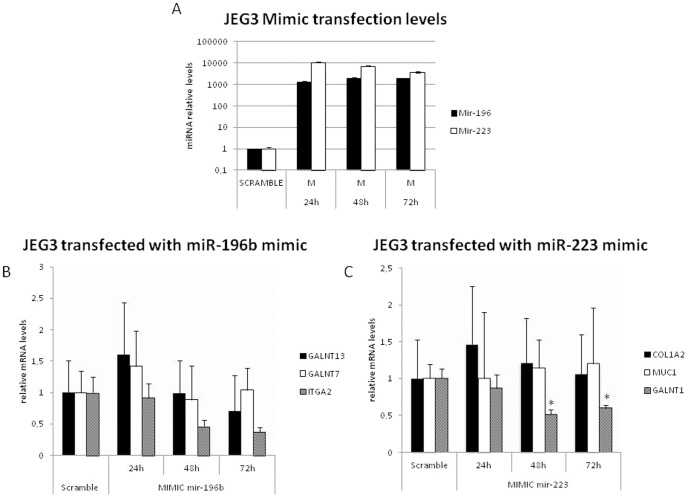
Trophoblast cell lines transfection with miR-196 and miR223. JEG3 cell line was used to check the inhibition effects of miR-196b and miR-223 mimics over predicted gene targets. miRNA expression levels were checked after mimic transfection by Real Time PCR and compared to scramble expression (A). JEG3 cells transfected with miR-196b mimic showed a sharp decrease of ITGA2 after 48 and 72 hours of transfection, while GALNT13 showed a slighter decrease (B). A significant ITGA2 expression was observed GALNT1 in JEG3 cells after 48 and 72 hours of miR-223 transfection (C). All data are presented as relative miRNA expression levels. *p-value <0.05.

To functionally prove that these miRNAs could have a direct effect on the repression of target genes, we transfected the human trophoblastic cell line JEG3 with miR-196b and miR-223 mimics and measured the gene expression of the predicted target genes (GALNT7, GALNT13 and ITGA2 for miR-196b; GALNT1, COL1A2 and MUC-1 for miR-223). The action of miR-196 mimic produced no significant changes in expression of the predicted target genes studied48 and 72 hours after transfection (Figure 4B). When we transfected JEG 3 line with miR-223 a significant downregulation of GALNT1 expression was observed with time. No downregulation of COL1A2 or MUC1 was observed. (Figure 4C).

## Discussion

To our knowledge this is the first study which has investigated the global differential miRNA repertoire of *in vivo* embryonic tissue from tubal EP patients using voluntarily terminated normal gestations as controls. Although we have investigated previously the Lin28/Let-7 system in ectopic and voluntary termination of pregnancy samples [25], a global miRNA approach has not been performed yet. We chose these controls because these pregnancies are viable, with a theoretically healthy growing trophoblast, thereby eliminating external factors such as toxics, chromosomal diseases, undiagnosed thrombophilias etc. which might lead to spontaneous abortion. In our microarray study, seven miRNAs were found to be differentially expressed, four (hsa-miR-196b, hsa-miR-30a, hsa-miR-873, and hsa-miR-337-3p) were downregulated and three (hsa-miR-1288, hsa-miR-451, and hsa-miR-223) were upregulated in EP pregnancy-derived tissues compared to VTOP samples. Although some studies have previously reported differential gene expression in the fallopian tubes of patients with EPs compared to intrauterine pregnancies [26], to date none of them have used embryonic tissue from VTOPs as controls.

Of these seven miRNAs, three of them (hsa-miR-196b, hsa-miR-223, and hsa-miR-451) were validated by real time PCR in a larger sample of EP and control tissues. These miRNAs might alter the downstream gene expression of dozens of genes, and thus investigating their gene targets may help us to elucidate the molecular mechanisms that intervene in ectopic pregnancy.

We are aware that we don't know the exact origin of these mRNA in our samples and could be very interesting to address this issue in future experiments. Ectopic and VTOP samples are complex tissues and the origin of these miRNAs could be important to understand the autocrine/paracrine effects of these miRNAs. Using bioinformatics tools, we analyzed all the predicted gene targets of these three miRNAs, obtaining the main pathways that could be altered in an ectopic pregnancy compared to an intrauterine gestation. Among these pathways, *mucin biosynthesis* was the most statistically significant. Two miRNAs (hsa-miR196b and hsa-miR223) are able to target three important genes in the mucin biosynthesis pathway: GALNT7, GALNT1, and GALNT13. These two miRNAs were found to be altered in EP tissues compared to controls, meaning that their expression could also be potentially altered in EP embryonic tissue, and therefore they were likely to be implicated in altering mucin expression. In fact, when we checked if GALNT13 gene expression was increased in EP samples, we found that it was upregulated as we had expected. It is noteworthy that not all the genes achieved significant differences in expression between both situations as expected. This is the case of GALNT7, GALNT1 and COL1A2, casting some doubts about the real implication of these pathways in vivo.

Although we saw some disparity in our results, some recent studies have reported deregulation of MUC1 and altered glycosylation in the tubal epithelium in EPs compared to controls [27], [28]. Furthermore, another recent study reports a MUC-1 expression deficiency in five recurrent EP patients, highlighting the importance of proper regulation of this O-Glycan biosynthesis pathway [29]. An additional statistically significant pathway found in our study was *extracellular matrix (ECM) receptor interactions*, with hsa-miR-196b and hsa-miR-223 able to interact with two targeted genes (COL1A2, and ITGA2); we also confirmed that integrin A2 (ITGA2) was significantly downregulated in EP samples.

Another study addressing tubal pregnancies showed that all trophoblastic cell types express matrix metalloproteinases (MMPs), and their regulators, tissue inhibitors of MMPs (TIMPs) [30]. These trophoblast cells express TIMP-1,-2,-3, MMP-2, and MMP-9 but in invasive trophoblast there is an increase in TIMP-1, -2, MMP-2, and MMP-14 which could explain the regulatory effects of differentially expressed miRNAs on trophoblast cell interactions with ECM components. This unique MMP and TIMP expression pattern at the feto-maternal interface, combined with our miRNA data, supports the regulation of these proteins by miRNA inhibition which could in turn alter trophoblast invasion during implantation and placentation, at least in EPs. More specifically, one of the downregulated miRNAs found in EP tissue, hsa-miR-196b, has also been described to be downregulated in endometrial stromal cells and seems to direct the suppression of c-myc and Bcl-2 mRNA expression as well as inhibiting proliferation and inducing apoptosis in these cells [31]. Interestingly, the hsa-miR-196 sequence is located on chromosome 7, and is encoded on an intron of the HOXA9 gene (Figure 3B). This is particularly intriguing given that HOXA9 is the main HOX gene that directs tubal formation during müllerian development (Figure 3C), that it is expressed in mouse and human adult oviduct tissue [32], and that hsa-miR-196 can directly inhibit the expression of HOXA9 in tumors [33].

We finally checked *in vitro* if the transfection of these miRNAs into trophoblast cell lines could alter the gene expression of these predicted gene targets. Although these miRNAs targets additional genes, we focused on the *mucin biosynthesis* and the *extracellular matrix (ECM) receptor interactions*. *In vitro* over-expression of these miRNAs triggered a clear downregulation of some predicted genes such as ITGA2 in the case of hsa-mi-196b and GALNT1 in the case of hsa-miR-223, but the expression of other predicted genes remains unaltered. This observation means that although some miRNA could directly alter the expression of predicted genes, the alteration of these proposed pathways is not completely demonstrated, requiring further research. We should note that miRNA target prediction software foretells ‘theorical’ gene targets with different ranges of probability, but usually these predictions do not match with real data [34]. Also, miRNAs usually have multiple target genes (sometimes, even more than 1000 genes). Then, miR-196b and miR-223 could be targeting other unexpected/undescribed genes, being some of them responsible of regulating genes implicated in these reported pathways, finally balancing the gene expression of our selected genes. For these reasons, target prediction could be a good first approximation, but sometimes, as we have seen, fails to identify (directly or indirectly) real target genes. Many of these targets have not been checked before, and deserve further research, maybe using luciferase assay or other techniques to show direct miR targeting. This unexpected discrepancy could be also partly due to the use of immortalized cells lines, instead of primary trophoblast cells. Sometimes, these types of cell lines do not behave as primary or tissue-derived cells. We think it is necessary to explore the real effects of these two miRNAs have in the global expression levels of these trophoblastic cells to find what pathways are really affected by this specific miRNAs.

In summary, we have discovered a clear differential pattern of miRNA expression in embryonic tissues derived from normal and eutopic/ectopic pregnancies, and have pinpointed some possible pathways that could be implicated in the processes of implantation and early placentation, although the real implication of these pathways in the ectopic pregnancy is yet to be determined.

## Supporting Information

Figure S1 **Real time PCR miRNA microarray validation in ectopic pregnancies and controls.** The same 8 VTOP and 8 EP embryonic samples that were used in the microarray experiments were used to validate by Real Time PCR the miRNA array. We observed a very significant decrease (p<0.001) in the EP tissue samples compared to VTOP samples for hsa-miR-196b (A) expression, while hsa-miR-223 (B) showed a significant (p<0.05) increase compared to VTOP controls. All data are presented as relative miRNA expression levels. *p-value <0.05; **p-value <0.01; ***p-value <0.001. Box plots represent the first quartile, median and third quartile; error bars show maximum and minimum relative expression levels.
(TIF)
