# Supplementary figures and images for: Embryonic miRNA Profiles of Normal and Ectopic Pregnancies
Source: PLoS One. 2014 Jul 11;9(7):e102185. doi: 10.1371/journal.pone.0102185 (PMC4094496; doi:10.1371/journal.pone.0102185)

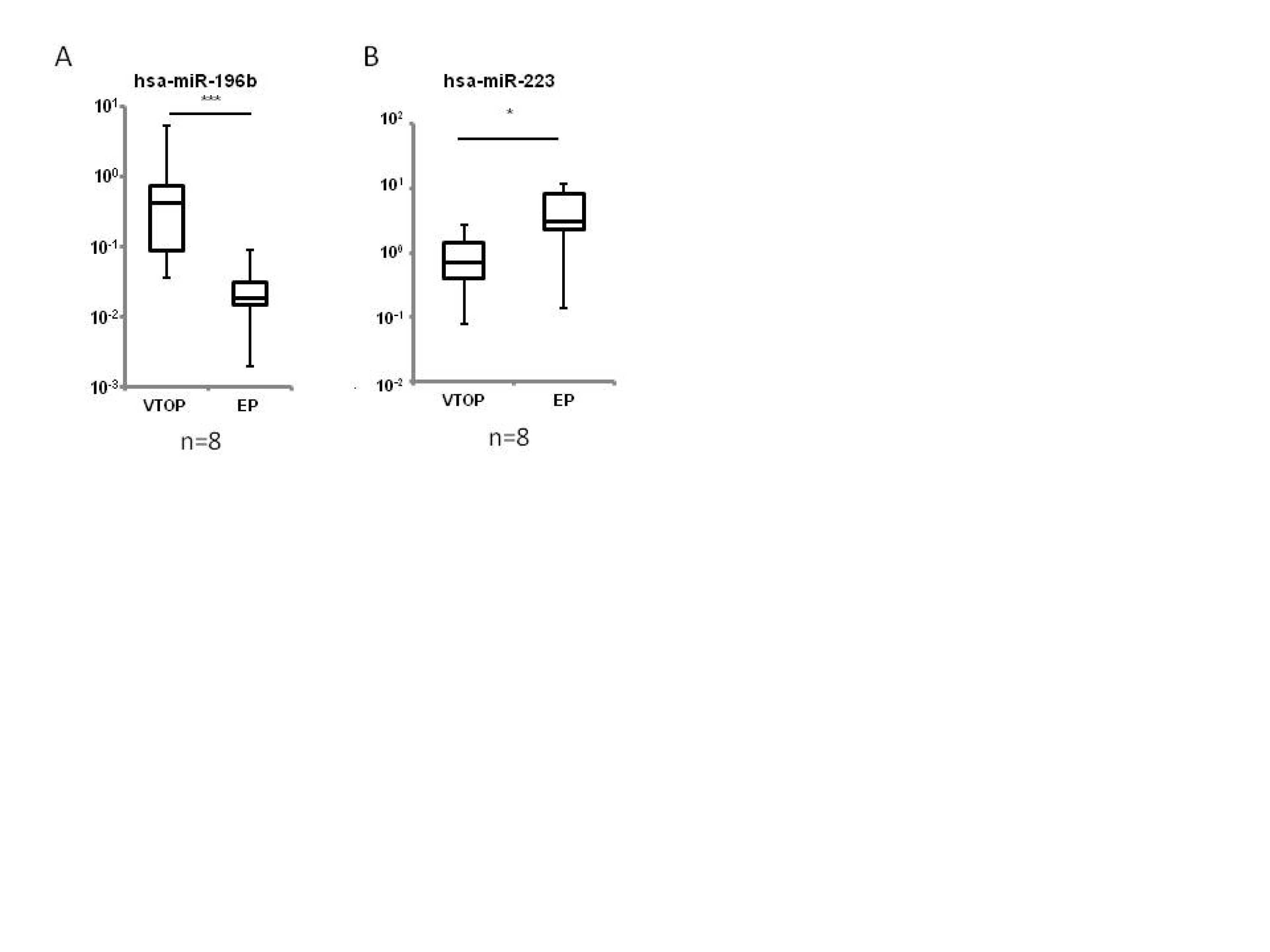

Supplement: Figure S1 — Real time PCR miRNA microarray validation in ectopic pregnancies and controls. The same 8 VTOP and 8 EP embryonic samples that were used in the microarray experiments were used to validate by Real Time PCR the miRNA array. We observed a very significant decrease (p<0.001) in the EP tissue samples compared to VTOP samples for hsa-miR-196b (A) expression, while hsa-miR-223 (B) showed a significant (p<0.05) increase compared to VTOP controls. All data are presented as relative miRNA expression levels. *p-value <0.05; **p-value <0.01; ***p-value <0.001. Box plots represent the first quartile, median and third quartile; error bars show maximum and minimum relative expression levels. (TIF) [file pone.0102185.s001.tif]
